# Nucleic Acid Aptamers Targeting Epigenetic Regulators: An Innovative Therapeutic Option

**DOI:** 10.3390/ph11030079

**Published:** 2018-08-24

**Authors:** Silvia Catuogno, Carla Lucia Esposito, Paola Ungaro, Vittorio de Franciscis

**Affiliations:** Istituto per l’Endocrinologia e l’Oncologia Sperimentale del CNR “G. Salvatore”, Via S. Pansini 5, 80131 Naples, Italy; silviacatuogno@libero.it (S.C.); carlaluciaespo@gmail.com (C.L.E.)

**Keywords:** epigenetics, aptamers, targeted therapy

## Abstract

Epigenetic mechanisms include DNA methylation, posttranslational modifications of histones, chromatin remodeling factors, and post transcriptional gene regulation by noncoding RNAs. All together, these processes regulate gene expression by changing chromatin organization and DNA accessibility. Targeting enzymatic regulators responsible for DNA and chromatin modifications hold promise for modulating the transcriptional regulation of genes that are involved in cancer, as well as in chronic noncommunicable metabolic diseases like obesity, diabetes, and cardiovascular diseases. Increasingly studies are emerging, leading to the identification of specific and effective molecules targeting epigenetic pathways involved in disease onset. In this regard, RNA interference, which uses small RNAs to reduce gene expression and nucleic acid aptamers are arising as very promising candidates in therapeutic approach. Common to all these strategies is the imperative challenge of specificity. In this regard, nucleic acid aptamers have emerged as an attractive class of carrier molecules due to their ability to bind with high affinity to specific ligands, their high chemical flexibility as well as tissue penetration capability. In this review, we will focus on the recent progress in the field of aptamers used as targeting moieties able to recognize and revert epigenetics marks involved in diseases onset.

## 1. Introduction

**Figure 1:**
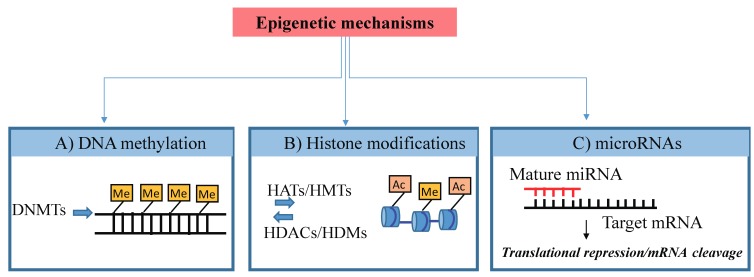
Scheme of Epigenetic mechanisms. Epigenetic mechanisms: (**A**) DNA methylation (Me) of cytosine residues within gene sequences that leads to transcriptional silencing. (**B**) Histone modifications include methylation (Me) and acetylation (Ac) that can lead to either activation or repression of gene transcription. (**C**) MicroRNAs are small RNAs able to regulate gene expression by paring to target mRNA sequences inducing target degradation or inhibition of translation. DNMTs, DNA methyltransferases; HATs, histone acetyltransferases; HDACs, histone deacetylases; HMTs, histone lysine methyltransferases; HDMs, histone lysine demethylases.

The term “epigenetics” defines processes regulating reversible changes in gene expression that occur without modifications in the DNA sequence [1]. Epigenetic marks (schematized in Figure 1) are generally heritable through mitosis across generations and determine whether a gene is silenced or expressed throughout the whole life course.

The best described epigenetic processes are DNA methylation, being the addition of methyl groups to DNA, post-translational modifications (PTMs) to histone proteins and, recently, a variety of noncoding RNAs (ncRNAs) amongst which microRNAs (miRNAs) are included, which bind to and regulate the expression of numerous mRNAs [2]. These epigenetic processes determine whether or not a particular gene is active in a given cell at a given time by changing its chromatin architecture and/or access by transcription factors (TFs) to its regulatory region. In contrast to the genome, which is largely stable, the epigenome may change during development or in postnatal life due to environmental influences. Indeed, epigenetic mechanisms may mediate the effects of exposure to a range of nutritional and environmental factors [1] and act as determinants of later health outcomes [3].

Although genome-wide studies have identified an elevated numbers of loci associated with complex human diseases, the causality of these pathologies remain unexplained [4]. Over the last decade, there has been increasing evidence of the role of epigenetic regulation in the development and progression of several chronic and severe diseases, including diabetes, cancers, and cardiovascular disorders [5,6]. Therefore, epigenetic processes, as those involved in DNA methylation and chromatin modification, have thus becoming promising therapeutic targets and precise prognostic markers. On the other hand, because of their high specificity of target recognition, RNA-based drugs (si/miRNAs, ASO, gRNAs, and aptamers) are emerging as extremely effective to edit genetic alterations, modulate epigenetic alterations, or directly interact with target enzymes.

Nucleic acid-based aptamers are a new class of high affinity and specific ligands, as well as potential antagonists of disease-associated proteins. In addition, they also provide effective delivery carriers for therapeutic diffusion of secondary reagents throughout the target tissues, thus reducing unwanted off-target effects.

In this review, we highlight recent reports on the development of aptamers as targeting agents for epigenetic marks underlining their potentiality as precise therapeutics. Such potentiality is further underscored by sophisticated reports that have recently shown that aptamers may serve to investigate the molecular mechanisms of epigenetic regulation.

## 2. Overview of the Epigenetics’ Processes

### 2.1. DNA Methylation

DNA methylation is a covalent modification that consists on the addition of a methyl group at cytosine of the DNA template. In mammals, DNA methylation occurs primarily at CpG dinucleotides. CpG are generally unmethylated at CpG island located in the promoter regions of many genes and normally methylated when scattered throughout the genome where they regulate transcription elongation and alternative splicing. In general, hypermethylation or hypomethylation of CpG islands at promoter regions are associated with repression or expression [7]. DNA methylation regulates several key physiological processes, including genomic imprinting, X chromosome inactivation, and stem cell plasticity.

In eukaryotes, the DNA methyltransferases (DNMTs) are responsible for transferring a methyl group from S-adenosylmethionine (SAM) to the 5’ carbon of cytosine, often in CpG dinucleotides. The human genome encodes five *DNMT* related genes but only three of them, named *DNMT1 DNMT3A*, and *DNMT3B*, are canonical cytosine-5 DNMTs that catalyze the addition of methyl groups to genomic DNA; (DNMT)3A and DNMT3B mediate de novo DNA methylation, whereas DNMT1 acts as a maintenance methyltransferase [8,9]. By contrast, DNMT2 and DNMT3 family members do not possess catalytic DNMT activity. 

DNA methylation is not stable; indeed, it has been recently shown that genomic DNA undergoes active demethylation yielding multiple intermediate forms of CpG modification, including hydroxymethylation, formylation, and carboxylation, suggesting that DNA methylation is a dynamic process [8,10]. The process occurs during zygote formation and in primordial germ cells, and in different types of somatic cells, including neurons and T lymphocytes.

### 2.2. Post-Translational Modifications of Histones

Within the eukaryotic cell nucleus, DNA is associated with histone proteins, resulting in a highly conserved structural polymer, referred to as chromatin [11]. Chromatin is composed of fundamental units called nucleosomes. A single nucleosome consists of a protein center, formed by eight histone proteins, two H2A, two H2B, two H3, and two H4 histones, and DNA is wrapped around this protein center. The addition of other proteins, such as the linker histone H1, coils the primary conformation into 30 nm fibers or higher-order chromatin structure. It is now widely accepted that chromatin is not a static structure but a dynamic entity intimately involved in the regulation of a variety of cellular functions [10,12]. Indeed, the control of gene expression is regulated primarily through modifications of the histone proteins controlling the packaging of DNA and therefore gene transcriptional regulation. The post-translational modifications of histones play key roles in generating the dynamic state of chromatin and include acetylation, biotinylation, methylation, phosphorylation, ubiquitination, SUMOylation, and ADP ribosylation that are in general finely tuned, reversible, and transient in order to meet the physiological needs of cells [13]. These modifications often occur on lysine, arginine, and serine residues and compose the “histone code”, which influences chromatin condensation and gene transcription [13]. Such modifications can distinguish key regulatory regions on the genome, including promoters, enhancers, gene bodies, and repetitive elements [14,15]. There is good evidence that transcriptionally active gene promoters are associated to acetylation of histone H3 and H4 together with methylation of lysine 4 residue on histone H3. By contrast, H3K9me3 (trimethylation on Lys 9 of histone 3) and H3K27 me3 (trimethylation on Lys 27 of histone 3) are two histone marks with a repressive role on gene promoters [14,15]. H3K4me1 (monomethylation of histone H3 at lysine 4) and H3K27ac (acetylation of histone H3 at lysine 27) are generally present in enhancer elements [16,17]. Histone modifications are mediated by several enzymes, such as histone acetyltransferases (HAT) (p300 and CREB-binding protein), histone deacetylases (HDAC) (HDAC1-11 and sirtuins), histone lysine methyltransferases (HMTs), and lysine demethylases (HDMs). These enzymes function by using as cofactors metabolites, such as acetyl-coA (HATs), S-(5’-adenosyl)-L-methionine (HMTs), and alpha-ketoglutarate (HDMs). Therefore, their misregulation can lead to metabolic abnormalities [18].

### 2.3. Noncoding RNAs

Numerous recent reports have demonstrated that also noncoding RNAs (ncRNAs) function as epigenetic regulators by modulating the expression of genes through transcriptional and post-transcriptional mechanisms [19].

Long noncoding RNAs (lncRNAs) are noncoding transcripts longer than 200 nucleotides that show variegated mechanisms of action and multiple functional activities. It is known that different lncRNAs regulate the epigenetic status of the human genome and are thus involved in the development of many human diseases, including cancer [20]. The mechanisms of action can be different. Some lncRNAs are able to alter chromatin conformation, other can recruit epigenetic regulators into specific genomic loci, and other can function as enhancer molecules.

MicroRNA (miRNAs) are the best-characterized family of small ncRNAs, about 19–24 nucleotides in length and highly conserved throughout organisms [21,22]. They regulate gene expression by binding to the 3’-untranslated region (UTR) after having recruited a protein complex, called RISC (RNA-induced silencing complex) to the target messenger RNA resulting in degradation or translational inhibition [23].

MiRNAs in metazoans do not need to form a perfect base-pair match to their target site, and thus it regulates over half of the human protein-coding genes [24,25]. Many studies indicated that aberrant expression of miRNA correlates with multiple diseases including obesity, cancer, cardiovascular disease, and diabetes [26]. As for other genes, their expression is under epigenetic controls, such as DNA hypermethylation of CpG islands within their promoter region [27,28,29].

Moreover, recently it has been reported thousands of cases of miRNA editing playing an important role in the progression of cancer pathogenesis [30]. The mechanism is mediated by adenosine deaminase acting on RNA (ADAR) enzymes that in humans convert adenosine (A) to inosine (I). MiRNA editing enables time- and location-specific regulation of mRNA expression, influencing various cellular properties and cancer development. As such, the dynamics of miRNA editing participates in the process of tumor progression and spreading, representing a new class of cancer epigenetic marks.

## 3. Aptamers

### 3.1. Aptamer Production

**Figure 2:**
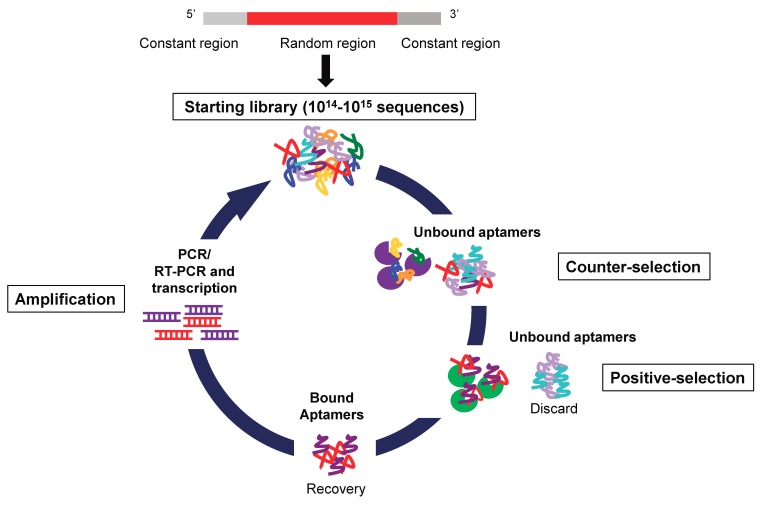
Schematic representation of SELEX technology. The starting RNA/DNA aptamer library is a high complexity library containing a variable region flanked by two constant regions for PCR amplification. The procedure involves reiterated rounds of: 1. Incubation of the library with nontarget molecules (counter-selection). 2. Incubation of unbound aptamers with targets (positive-selection step). 3. Recovery of bound aptamers. 4. Amplification of the bound aptamers by PCR (for DNA library) or RT-PCR and transcription (for RNA library).

Nucleic acid aptamers are short single stranded DNAs or RNAs that, by folding into particular three-dimensional shapes, bind with high affinity (nanomolar-picomolar range) and specificity a specific target molecule. Aptamers are selected through an in vitro technique of combinatorial chemistry named Systematic Evolution of Ligands by Exponential enrichment (SELEX) (Figure 2), first described in 1990 by two independent laboratories [31,32].

Of note, the first description of the SELEX procedure was specifically designed to deeply understand the molecular mechanisms underlying the regulation of gene expression [32]. At that time, Tuerk C and colleagues were studying the eight loop nucleotides within the hairpin of the translational operator of the bacteriophage T4 gene 43 mRNA. To this end, they mutated completely the hairpin loop within that motif giving rise to a small library of 4^8^ (approximately 65,000) different eight nucleotides sequences that revealed to be sufficient to evolve high affinity ligands for gene 43 protein (the T4 DNA polymerase). Since then, this technique has been used to select aptamers against a wide range of molecules and chemical compounds. In many cases, the SELEX approach has been used to select aptamers against cell proteins overexpressed or mutated in pathological states. In these cases, purified proteins (protein-SELEX) [33,34,35,36] or whole living cells (cell-SELEX) [37,38,39,40] can be used as targets.

### 3.2. Aptamer Advantages as Diagnostic and Therapeutic Tools

**Figure 3:**
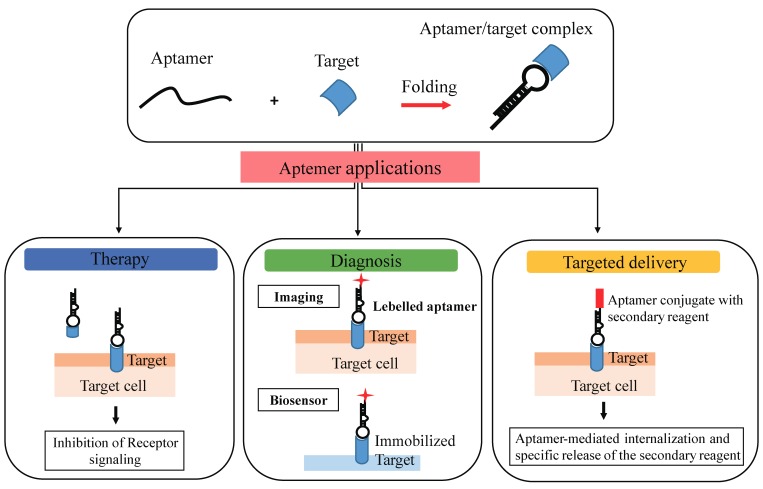
Aptamer applications. Aptamers bind with high affinity and specificity to their target molecules by folding in tridimensional structures and show great potential as therapeutic, diagnostic, and delivery tools.

Aptamers are also termed “nucleic acid antibodies” and couple the advantages of antibodies, relatively to the biological function, to that of nucleic acids, essentially deriving from their chemical nature. They show a number of important advantages over antibodies. Importantly, the SELEX method used for selection avoids the use of animals and shows a very high batch fidelity. Further, they possess a small size, they are not immunogenic, they can be easily chemically modified to improve serum stability, pharmacodynamics, and pharmacokinetics [41,42,43]. Another important property of aptamers is that they have been reported to be nonimmunogenic and nontoxic. This is based on the fact that the Toll-like receptors-mediated innate immune response against exogenous nucleic acids, is abrogated by modified nucleotides containing aptamers. In addition to the specific target binding, it has been demonstrated that many aptamers against cell surface receptors, upon binding to their target, are able to inhibit downstream molecular pathways, thus modulating cellular processes associated with cancer development, inflammatory diseases, viral infection, cardiovascular illness, and other human pathological states. Moreover, different aptamers against cell surface receptors demonstrated the capacity to internalize in a receptor-mediated manner and have been successfully developed as carriers for the selective delivery of many therapeutic drugs, including chemotherapeutics, toxins, and therapeutic oligonucleotides (siRNAs, miRNAs, or antimiRs) for effective targeted therapy [44]. Therefore, for their unique characteristics, aptamers represent effective diagnostic and therapeutic tools for a wide range of human pathologies, as well as for the identification of new interesting markers (Figure 3).

## 4. Aptamer-Based Targeting of Epigenetics Marks

Aptamers may be used for direct targeting of epigenetic players or as delivery carriers of secondary reagents involved in the epigenetic processes. So far, there are very few examples of aptamers selected against molecules regulating the epigenetic marks. However, one of the advantages of aptamers is that they can be selected to bind and inhibit any target of interest.

### 4.1. Aptamer-Mediated Targeting of Epigenetic Players

Histone PTMs are involved in the regulation of many cell processes, such as RNA transcription and DNA replication and repair, and are thus associated with different human diseases. Anyway, many efforts are still needed for a complete and deepen comprehension of their function. The high complexity and dynamic changes of histones’ PTMs makes it difficult to identify them comprehensively. So far histone purification is performed with very complicated and expensive methods, which use HPLC or antibody-based strategies followed by acid or salt-extraction [45,46,47,48]. Hence, fast, sensitive, and effective methods for the extraction of histone are highly desirable.

Until now, aptamer-based analytical methods have been created to bind virtually any target including ions, small molecules, drugs, peptides, proteins, and even whole cells [49]. Unfortunately, only some aptamers recognize, with high affinity and specificity, histone post-translational modifications. Because histones are highly positively charged proteins, using nucleic acid aptamers to specifically recognize different histone classes may be a very challenging objective. To this end, Lin L and colleagues used as a target a histone H4 small peptide with a pI = 12.00 and adopted an in vitro selection protocol that relies on capillary electrophoresis (CE) to separate functional aptamers from unbound sequences [50]. The starting nucleic acid library was incubated with the H4 peptide free in solution for 1 h at room temperature. Unbound sequences were separated and discarded, while bound DNA molecules were collected in a separate vial. At each round of selection, PCR was used to amplify the enriched DNA pool. Next, the DNA was purified and denaturized on streptavidin-coated beads to obtain DNA single-stranded. After four rounds of selection, a second peak became visible in the CE chromatogram, indicating that the pool had become enriched in aptamers with affinity to the H4 peptide. Two aptamers have been characterized showing high binding affinity towards the histone H4 peptide (dissociation constant (Kd) of approximately 5–10 nM). Most important, the selectivity of the two anti-H4 aptamers was as well determined by recognition imaging microscopy. Both recognized the recombinant H4, and at lower extents the histone H3, and at very low levels of recognition also H2A and H2B proteins, paving the way to further developments [50]. The same group adopted the CE-based SELEX to generate aptamers targeting a histone PTM [51]. In order to target the histone H4 acetylated at lysine 16 (H4-K16Ac) they used a short peptide spanning the N-terminal tail of H4 where the PTM is located. To remove molecules that bind to the unmodified histone H4 (H4-K16) they introduced, at each round, a negative selection step using as target the unmodified N-terminal tail of H4 followed by a positive selection step. After four rounds of CE-SELEX one aptamer, named clone 4.20 (dissociation constant of approximately 47 nM), was further characterized. Authors compared binding of the aptamer (clone 4.20) to the H4-K16Ac with the unmodified H4-K16 and with the same peptide with acetylated lysine, but at position 8 instead of 16 (H4-K8Ac). Authors showed that the aptamer is highly specific for acetylated lysine in position 16, but much less (approximately 2400 times) for the nonacetylated peptide or a peptide with the acetylated lysine at position 8, revealing to be approximately 20 times more specific as compared to commercial antibodies [51].

**Figure 4:**
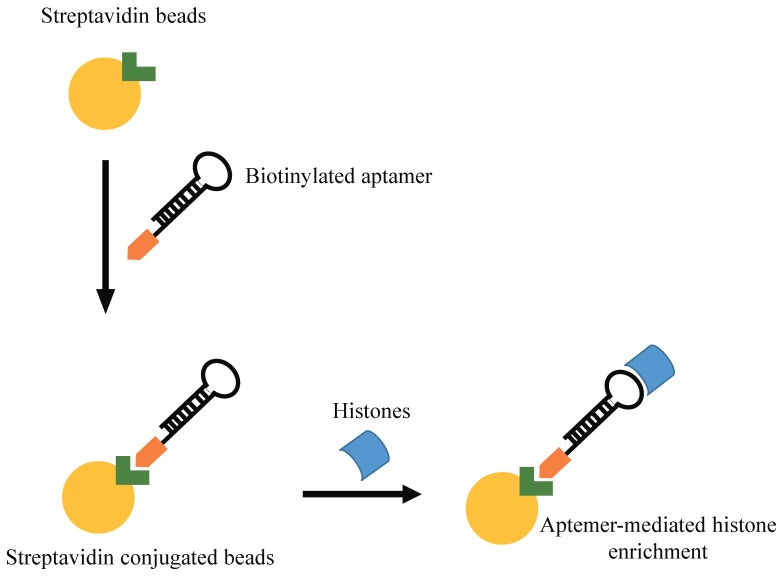
Aptamer-mediated histone enrichment. Scheme of the strategy used by Shao N et al. for histone enrichment by using aptamers.

Shao N et al. proposed an innovative aptamer-based approach for histone protein enrichment, based on the recognition of the N-terminal tail of the histone core as a target. In order to prepare a modified spin column for enrichment of histones, these authors selected an aptamer recognizing histone H4 peptide with acetylation at K16 residue. In addition to its strong affinity for histone H4, this column revealed to have a general good binding capacity for all histones, and was thus used for their extraction in Hela cells (Figure 4) [52]. Further, a SELEX approach has been performed against a modified histone H3 N-terminal peptides (H3R8Me2sym) [53]. The authors used a biotinylated 14-amino acid mimic peptide of this modified histone tail as target. After 10 rounds of SELEX, they identified an aptamer with low nanomolar binding affinity (K(d) = 12 nM) for the H3 peptide.

### 4.2. Aptamers as Delivery Carriers

**Figure 5:**
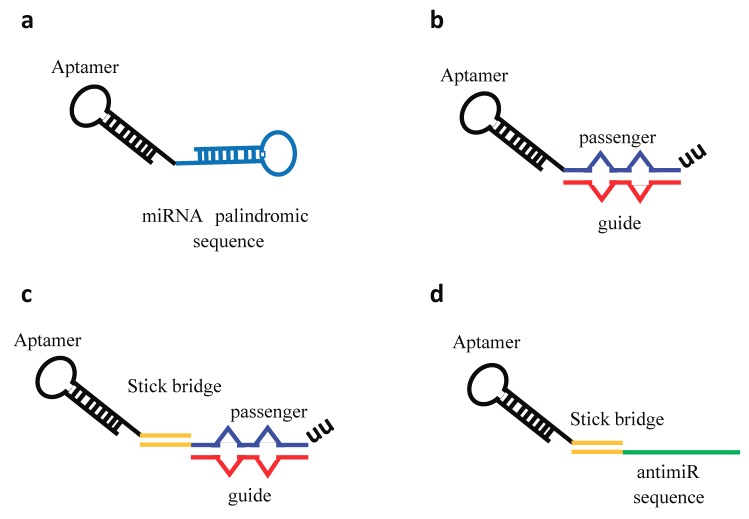
Aptamer–miRNA/antimiR conjugates. Schematic representation of different conjugation strategies adopted. (**a**) Aptamer covalently conjugated to a miRNA palindromic sequence. (**b**) Aptamer directly conjugated with the miRNA passenger strand followed by the annealing of the miRNA guide strand. The miRNA duplex contains internal partial complementarity and increased length extension (27/26 bases) to obtain a more effective Dicer substrate. (**c**) Stick-based approach in which the aptamer and the miRNA moieties are connected through the annealing of complementary GC-rich stick sequences. (**d**) Stick-based approach in which the aptamer and the antimiR are connected through the annealing of complementary GC-rich stick sequences.

More widely reported is the use of aptamers for the specific delivery of secondary reagents and many studies have described the effective aptamer-mediated delivery of miRs or anti-miRs molecules by using different molecular structures of conjugates [44] (Figure 5).

Recent reports demonstrated the important role of miRNAs in aberrant mechanisms of DNA hypermethylation in malignant cells [54]. As an example, miR-29b expression in acute myeloid leukemia cells reduced the expression, at both RNA and protein levels, of DNA methyltransferases *DNMT1*, *DNMT3A*, and *DNMT3B*. The downregulation of *DNMT3A* and *DNMT3B* is the result of a direct interaction between miR-29b and the 3’ untranslated regions of these genes, while the decrease of *DNMT1* expression was reached indirectly via downregulation of *Sp1*, a known transactivating factor of the *DNMT1* gene. In tumor cells, the reduced expression of DNA methyltransferases restored phosphatase and tensin homolog deleted on chromosome ten (*PTEN*) gene expression. Importantly, growing evidences support a role for methylation as the main factor causing low *PTEN* expression in several tumors [55,56]. As an example, in ovarian endometrioid carcinoma and clear cell carcinoma of the ovary, inactivation of the *PTEN* gene through methylation to its promoter region is an early event in the development of the disease [57,58]. 

Thus, overexpression of miR-29b could be considered a strategy to upregulate *PTEN* gene expression through a hypomethylation machinery.

Dai F and coauthors [59] were the first to develop a chimera composed of anti-mucin 1 (MUC1) DNA aptamer and miR-29b and tested its delivery and functions in epithelial ovarian adenocarcinoma cells. They demonstrated that the chimera effectively downregulated *DNMT* expression, induced hypomethylation of *PTEN* promoter, upregulated *PTEN* expression, and subsequent cell apoptosis. Mucin 1 (MUC1) is a cell surface glycoprotein that is overexpressed and aberrantly glycosylated on the cell surface of a majority of human adenocarcinomas [60]. The chimera of MUC1 aptamer and miR-29b can be considered an amazing strategy for a tumor tissue-specific targeting therapy of ovarian cancer. However, more studies are needed to validate its specificity and also to reduce the possible toxicity to normal tissue.

In addition to miR-29b, more comprehensively, aptamers have been used for the selective delivery of different miRNAs and single stranded antimiRs. For example, Liu N and colleagues [61] used the anti-MUC1 aptamer for the selective delivery of let-7i miRNA to paclitaxel resistant OVCAR-3-cells and demonstrated the ability of the aptamer-based conjugate to reverse the resistant phenotype.

More recently, a 2’-F-pyrimidine modified RNA aptamer targeting Axl receptor (GL21.T aptamer) has been used to selective deliver miR-212 into human non-small-cell lung carcinoma (NSCLC) Axl+ cells [62]. The aptamer was covalently conjugated with miR-212 to generate a unique chimeric molecule able to drive selective miR-212 internalization. In vitro treatment produced clear miR-212 upregulation and *PED* downregulation, consequently leading to TRAIL sensitization. The same aptamer has been also successfully used for the selective delivery of the tumor suppressor let-7g miRNA in NSCLC, and the selectivity and the effectiveness of the system was proved both in vitro and in vivo [63].

Interestingly, Catuogno S. and colleagues [64] described an aptamer-based approach for the selective delivery of single strand antimiRs in tumor cells both in vitro and in vivo. In this work, the GL21.T aptamer and an anti-Platelet-derived growth factor receptor (PDGFR)β aptamer, named Gint4.T, were noncovalently conjugated through a stick-based approach with antimiR sequences. The procedure consisted in the extension of the 3’ end of both aptamer and antimiR with a “stick” tail (17-mer long) allowing their stable annealing without altering aptamer folding. The generated chimeric conjugates were validated both in vitro and in vivo for the selective antimiRs delivery in glioblastoma (GBM).

Further, GL21.T and Gint4.T aptamers were conjugated to both miRNA and antimiR sequences through the same stick-based approach, in order to investigate the effectiveness of combined treatments for efficient GBM eradication [65].

### 4.3. Aptamer-Based Biosensors 

MicroRNAs play pivotal roles in many human pathologies, therefore microRNA detection has demonstrated to be useful in disease diagnosis. For this purpose, different biosensor-based techniques, showing good sensitivity and specificity as well as multiplexing capabilities, have been developed [66]. Interestingly, aptamer-based biosensors have been also developed. Pang X and coauthors proposed a photoelectrochemical (PEC) aptasensor in which CH3NH3PbI3 quantum dots (QDs) sensitized ZnO nanosheets (NSs) were used for detection and quantitative determination of miR-155. The proposed biosensor showed a very high sensitivity, good selectivity, stability, reproducibility, and a low detection limit, thus offering the perspective of a potential applicability for miRNA biomarker detection in clinical diagnosis [67].

Another example of an aptamer-based sensor for miRNA detection was given by Wang and colleagues that developed an electrochemistry integrated aptasensor for miR-21 detection [68]. The system was based on a first hybridization step of an initiate sequence A and initiate sequence B that was perturbed by the presence of miR-21, allowing the replacement and the consequent release of the initiate B. In the following step, the released initiate B formed a Y-shaped DNA structure with two capture sequences on an electrode. The addition of two hairpins (aptamers) formed a Y-shaped branching ds-DNA structure that functions as a template for the synthesis of copper nanoclusters used for signal detection.

Mentioned examples demonstrate the emerging large utility of aptamers in the epigenetics field and their wide versatility that makes them useful tools for histone modification detection, miRNA/antimiR selected delivery and miRNA detection as well, opening the possibility to further develop aptamers for miRNA editing study.

## 5. SELEX-Based Strategies to Investigate Mechanisms of Gene Expression

In addition to the use of aptamers as targeting molecules, the great potentiality of the SELEX technology has been applied for genome editing studies in an innovative work from Yin Y et al. [69] The authors developed a methylation-sensitive SELEX to find out transcription factors (TFs) that prefer CpG-methylated sequences. They performed two parallel high-throughput SELEX (HT-SELEX) by using a collection of ~550 full-length TFs and a DNA binding domains libraries either unmethylated or methylated using the CpG-specific cytosine 5-methylase M.SssI before each selection round. The post-SELEX analyses revealed that many developmentally important TFs preferentially bind to mCpG sequences, suggesting that DNA methylation is a key aspect for TF binding. In addition, by structural analyses, the authors demonstrated the dependence of methylcytosine specificity from hydrophobic interactions with the methylcytosine 5-methyl group.

Aptamer potentiality was further demonstrated in a recent paper by Zaghlool S.B. et al. [70] reported the use of a multi-omics association study to investigate the correlations of previously identified CpG sites with a set of ~4000 deep molecular phenotypes. Among other strategies, they used aptamer technology (SOMAscan plataform, Somalogic Inc., Boulder, CO, USA) [71] to quantify a total of 1124 proteins in 356 plasma samples. The described approaches underline a great potential of the SELEX technology and open new innovative field of application.

## 6. Conclusions

The present review discusses some original examples of aptamer applicability in the epigenetic field. Thanks to their features, aptamers represent promising tools for both therapeutic and diagnostic purposes as well as targeted delivery. One key factor is aptamer high affinity and specificity that offers a great potential for epigenetic targeting. Indeed aptamers may distinguish even very close targets that differ only by a few functional groups [72,73]. This aspect is of fundamental impact since it opens the possibility to develop high selective molecule able to discriminate even single epigenetic modification.

While the use of aptamers for miRNA selective delivery has grown rapidly in the last years with several examples, their application to direct epigenetic targeting is still at an early stage. However, the reported examples underline how aptamers hold a great potentiality in the field, for both diagnostic and therapeutic purposes and could bring a new era to the epigenetic research.

## 7. Future Perspectives

The implication of epigenetic regulation in the development of devastating diseases, mostly cancer and neurological disorders, underscores the potential of developing drugs able to revert disease-associated epigenetic marks. To date several drugs developed to target epigenetic modifiers are in active clinical trials, and so far, molecules targeting DNA N-methyl transferase inhibitors (DNMTi) and histone deacetylase inhibitors (HDACi) have been approved by FDA, such as nucleoside-based compounds, 5-azacytidine and 5-aza-2’-deoxycytidine. However, the lack of selectivity, toxicity and chemical instability represent a serious concern for the use of these drugs.

The recent observations that the epigenetic control of gene transcription can rely on structural components within long noncoding RNAs [74,75] pave the way for new drugs able to trigger the selective DNA methylation pattern of aberrantly expressed genes. A paradigmatic example is provided by the finding that short folded structures within a lncRNA, named *ec*CEBPA, can bind to and inactivate *DNMT1* resulting in prevention of *cEBPA* tumor suppressor gene methylation [75]. In this regard, nucleic acid aptamers are folded RNA-based therapeutic that have the double advantage to “mimic” the structures of intracellular protein-binding ncRNAs combined with the high target selectivity and lack of toxicity. Owing to these properties, aptamers are promising candidates as precise and safe novel drugs with epigenetic modes of action. Further, the use of the SELEX technology to reproduce the functional/interacting sites of protein-binding oligonucleotides has revealed to be a promising innovative tool to investigate the impact of epigenetic mechanisms (i.e., cytosine methylation) on gene expression. Moreover, aptamer technology can be used as a powerful tool to investigate the link between CpG methylation and body’s response to disease or environmental stress.

## Abbreviations

The following abbreviations are used in this manuscript:

ADAR	adenosine deaminase acting on RNA
CE	capillary electrophoresis
DNMTs	DNA methyltransferases
DNMTi	DNA N-methyl transferase inhibitors
GBM	glioblastoma
HATs	histone acetyltransferases
HDACs	histone deacetylases
HDACi	histone deacetylase inhibitors
HMTs	histone lysine methyltransferases
HDMs	lysine demethylases
HT-SELEX	high-throughput SELEX
Kd	dissociation constant
miRNAs	microRNAs
MUC1	Mucin 1
NSs	nanosheets
ncRNAs	noncoding RNAs
NSCLC	non-small-cell lung carcinoma
PDGFR	Platelet-derived growth factor receptor
PTEN	phosphatase and tensin homolog deleted on chromosome ten
PTMs	post-translational modifications
QDs	quantum dots
RISC	RNA-induced silencing complex
SAM	S-adenosylmethionine
SELEX	Systematic Evolution of Ligands by Exponential enrichment
TFs	transcription factors
UTR	untranslated region
